# Mover Position Detection for PMTLM Based on Linear Hall Sensors through EKF Processing

**DOI:** 10.3390/s17040782

**Published:** 2017-04-06

**Authors:** Leyang Yan, Hui Zhang, Peiqing Ye

**Affiliations:** 1 Department of Mechanical Engineering, Tsinghua University, Beijing 100084, China; yly15@mails.tsinghua.edu.cn (L.Y.); wwjj@tsinghua.edu.cn (H.Z.); 2 State Key Laboratory of Tribology, Tsinghua University, Beijing 100084, China

**Keywords:** linear motor, position detection, linear Hall sensors, extended Kalman filter

## Abstract

Accurate mover position is vital for a permanent magnet tubular linear motor (PMTLM) control system. In this paper, two linear Hall sensors are utilized to detect the mover position. However, Hall sensor signals contain third-order harmonics, creating errors in mover position detection. To filter out the third-order harmonics, a signal processing method based on the extended Kalman filter (EKF) is presented. The limitation of conventional processing method is first analyzed, and then EKF is adopted to detect the mover position. In the EKF model, the amplitude of the fundamental component and the percentage of the harmonic component are taken as state variables, and they can be estimated based solely on the measured sensor signals. Then, the harmonic component can be calculated and eliminated. The proposed method has the advantages of faster convergence, better stability and higher accuracy. Finally, experimental results validate the effectiveness and superiority of the proposed method.

## 1. Introduction

Permanent magnet tubular linear motors (PMTLM) have been the focus of significant research for the last few decades due to their high power density, small end effect and excellent dynamic performance [1,2]. PMTLMs have been increasingly applied in electromagnetic launches, healthcare and other applications with strict volume restriction [2].

Mover position detection is one of the key techniques for servo control of PMTLM, and is vital for thrust control and positioning accuracy. In most applications, a grating scale is the most commonly used position sensor [3,4]. A grating scale has the advantages of high precision, high resolution and fast response, but also suffers from shortcomings like high cost and large volume. Furthermore, an auxiliary guide is needed for a grating scale, which restricts its application. Some researchers have also adopted sensorless methods [5,6,7,8,9,10], utilizing the voltage and current information to estimate mover position based on the electrical PMTLM model. Sensorless methods can be divided into two categories: back-EMF-based methods and high frequency injection methods. The former is highly dependent on electrical parameter accuracy and shows bad performance in low-speed region, while the latter introduces thrust ripple and extra loss. Generally, sensorless methods are unfit for applications where high precision is demanded.

As an alternative, linear Hall sensors are more and more widely used nowadays to detect mover position [11,12,13,14,15,16,17]. They have merits like low cost and high robustness, and the installation is easy to implement. Two linear Hall sensors electrically separated 90 degrees are widely adopted for both rotor angular detection and mover position detection. Linear Hall sensors can measure varying magnetic field strength and continuously output the corresponding sine and cosine wave signals. However, Hall sensor signals are contaminated by third-order harmonics, which are caused by air-gap magnetic distortion [18]. Harmonics disrupt position detection accuracy, and should be filtered out from Hall sensor signals. Some attempt to minimize the effect of harmonics by optimizing the installation position of Hall sensors [11,12,13]. It has been found that the harmonic component is diminished as the sensors are radially away from the permanent magnet (PM), but the signal amplitude also decreases. Others utilize signal processing methods to remove harmonics [14,15,16,17]. In [14], a phase-lock loop (PLL) is employed. A PLL contains a PI-type low-pass filter, which can filter out high-order harmonics. But the bandwidth of a PLL is small, and its performance deteriorates in low-speed regions. To tackle this problem, an adaptive notch filter (ANF) is adopted to remove the third-order harmonics before applying a PLL [15]. The notch frequency is adaptively tuned by software, so the ANF operates well even when the speed is very low. In [16], an improved ANF, i.e., a synchronous frequency extractor (SFE), is presented. Compared with an ANF, an SFE can filter out higher order harmonics. Therefore, the position detection error is reduced even more, as the 5th and 7th order harmonics are also removed. Though ANF-based processing methods exhibit excellent performance in filtering out signal harmonics in all speed regions, their convergence rate and stability are not satisfactory, which could be severe problems in PMTLM application.

In [19,20], an extended Kalman filter (EKF) processing method is used to remove random errors in rotor angular detection of PMSM. With an extremely simple form, an EKF can effectively eliminate white noise in Hall sensor signals.

In this paper, EKF is utilized to filter out third-order harmonic components from Hall sensor signals. The amplitude of fundamental component and the percent of harmonic component are estimated online via an EKF model. In this way, the harmonics can be observed and removed. Due to the merits of an EKF, both the dynamic and static accuracy of mover position detection can be greatly improved in all speed regions, while the convergence rate and stability are also guaranteed.

Section 2 describes the implementation of mover position detection based on linear Hall sensors. In Section 3, the ANF processing method is described, and its stability is analyzed. In Section 4, EKF model is constructed, and then the idea of EKF processing is presented. Finally, Section 5 validates the feasibility of the proposed method via experiments.

## 2. Position Detection Based on Linear Hall Sensors

**Figure 1:**
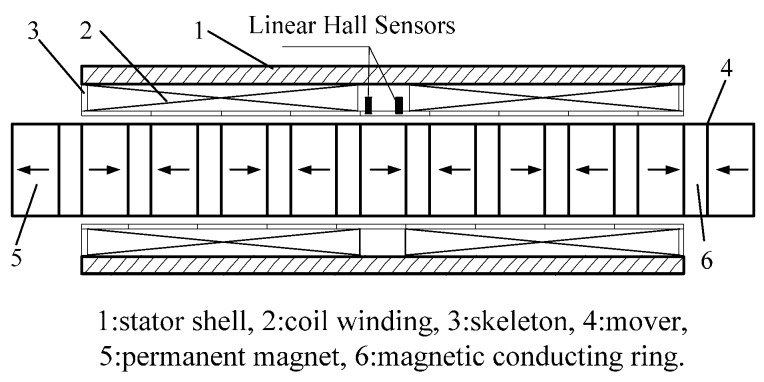
Diagram of PMTLM structure.

**Figure 2:**
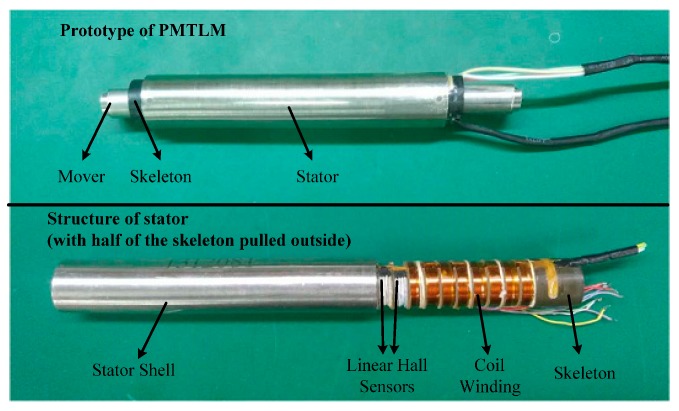
Physical map of a PMTLM.

Linear Hall sensors are based on the Hall Effect, and the sensor output is linear to the magnetic field strength. As the mover transports inside the stator, the air-gap magnetic field is approximately distributed in a sine wave. The diagram and physical map of PMTLM used for experiments are shown in Figure 1 and Figure 2. The pole pitch of PMTLM is 10 mm, and the two Hall sensors are separated by 5 mm axially so that the two sensor signals are electrically 90° apart. The Hall sensors continuously output position-dependent sine/cosine signals, and then the mover position can be obtained by using an arctangent function, as shown in Equations (1) and (2).

(1) $$u_{ak}=u_{mk}\sin\theta_{k},\;u_{bk}=u_{mk}\cos\theta_{k}$$

(2) $$\begin{array}{c} \theta_{k}=\mathrm{atan}2\left(u_{ak},u_{bk}\right)\equiv \left\{ \begin{array}{l} \arctan\left(u_{ak}/u_{bk}\right),\;if\;u_{ak}>0,u_{bk}\geq 0\\ \arctan\left(u_{ak}/u_{bk}\right)+\pi ,\;if\;u_{bk}<0\\ \arctan\left(u_{ak}/u_{bk}\right)+2\pi ,\;if\;u_{ak}\leq 0,u_{bk}\geq 0\; \end{array}\right.\\ x_{k}=\left(\tau \cdot \theta_{k}\right)/\pi \end{array}$$

where *x_k_* is mover position, *τ* is pole pitch, *u_ak_*, *u_bk_* are signals detected by Hall sensors, *u_mk_* is the amplitude of the fundamental component and *θ_k_* is the corresponding electrical angle of the mover.

**Figure 3:**
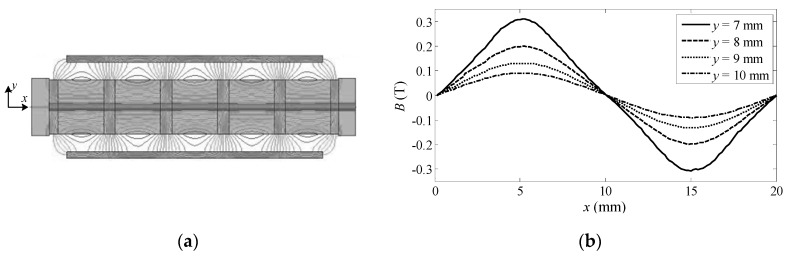
(**a**) FEM simulation result of magnetic line distribution; (**b**) Distribution curves of magnetic field strength at different radial distances.

Figure 3a is a 2-D finite-element-method simulation result by Ansoft Maxwell, demonstrating the distribution of a magnetic line in air-gap. The distribution curves of magnetic field strength at different radial distances can be seen more specifically in Figure 3b. Obviously, the distribution curve is not a pure sine wave, and is even approximate to a triangular wave when 7 mm away from PMs, indicating that the magnetic field contains large odd-order harmonics. As the radial distance becomes farther, the magnetic line becomes closer to a sine wave while its amplitude decreases. Limited by the size of the motor, the two Hall sensors are installed 9 mm away from PMs in practice, producing third-order harmonic components in Hall sensor signals, as shown in Equation (3).

(3) $$\begin{array}{c} u_{ak}=u_{mk}\left(\sin\theta_{k}-r_{k}\sin3\theta_{k}\right)\\ u_{bk}=u_{mk}\left(\sin\left(\theta_{k}+\pi /2\right)-r_{k}\sin3\left(\theta_{k}+\pi /2\right)\right)=u_{mk}\left(\cos\theta_{k}+r_{k}\cos3\theta_{k}\right) \end{array}$$

where *r_k_* is the percent of the third-order harmonic component.

Therefore, the raw electrical angle ${\hat{\theta }}_{k}$ calculated by Equation (2) contains a large error, as described in Equations (4) and (5).

(4) $${\hat{\theta }}_{k}=\mathrm{atan}2\left(u_{ak},u_{bk}\right)=\mathrm{atan}2\left(\sin\theta_{k}-r_{k}\sin3\theta_{k},\cos\theta_{k}+r_{k}\cos3\theta_{k}\right)$$

(5) $$e_{k}=\theta_{k}-{\hat{\theta }}_{k}\approx \tan e_{k}\approx r_{k}\sin4\theta_{k}\approx r_{k}\sin4{\hat{\theta }}_{k}$$

Apparently, the third-order harmonics in sensor signals would introduce a fourth-order error in position detection result. The amplitude of error is related to the percentage of the harmonic component, which could be large in some applications. Thus, it is necessary to filter out third-order harmonics from the original sensor signals.

## 3. Analysis of ANF-Based Signal Processing

In order to eliminate harmonics in Hall sensor signals, [15] proposed a signal processing procedure based on ANF, and an improved ANF is presented in [16]. To the best of the author’s knowledge, ANF remains one of the most effective signal processing methods to filter out harmonics. ANF is a notch filter that is constructed by a closed loop, and can operate well even in a low-speed region. In this section, its performance will be discussed.

**Figure 4:**
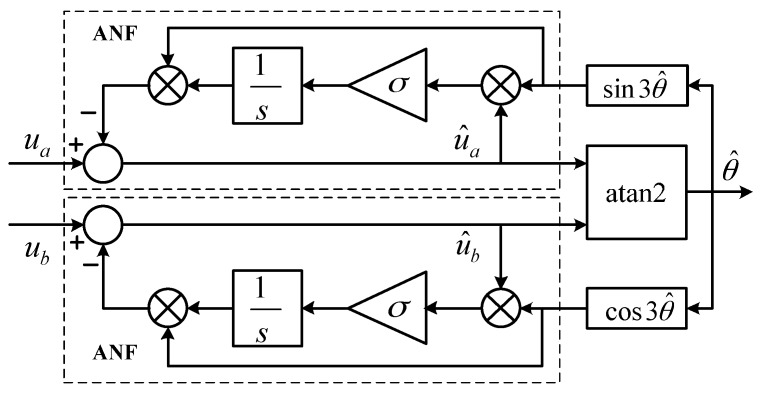
Diagram of ANF processing.

The simplified diagram of ANF processing is shown in Figure 4. Two ANFs are used to process two channel sensor signals. Take one ANF for example; the transfer function from *u_a_* to ${\hat{u}}_{a}$ is

(6) $$H(s)=\frac{{\hat{U}}_{a}}{U_{a}}=\frac{s^{2}+\omega_{h}^{2}}{s^{2}+\sigma s+\omega_{h}^{2}}$$

Equation (6) has the same form as a conventional second-order notch filter. Its gain equals zero at *s = jω_h_*, so the harmonic component at the frequency of *ω_h_* is filtered out. The parameter *σ* determines the sharpness of the notch filter. As *σ* becomes larger, the bandwidth of ANF becomes larger, thus the convergence rate is faster, and yet the filtering performance gets worse. Hence, there is always a dilemma between faster convergence rate and better filtering performance for ANF.

Moreover, the ANF-based signal processing method is not stable at zero speed. In steady state, the inputs of the two integrators in Figure 4 should be fluctuating around zero. Particularly, all variables in Figure 4 are constant when mover speed is zero, and then the inputs of the two integrators are supposed to be equal to zero, i.e.,

(7) $$\begin{array}{l} \sigma \cdot {\hat{u}}_{a}\cdot \sin\left(3\cdot \mathrm{atan}2\left({\hat{u}}_{a},{\hat{u}}_{b}\right)\right)=0\\ \sigma \cdot {\hat{u}}_{b}\cdot \cos\left(3\cdot \mathrm{atan}2\left({\hat{u}}_{a},{\hat{u}}_{b}\right)\right)=0 \end{array}$$

Taking $\mathrm{atan}2\left({\hat{u}}_{a},{\hat{u}}_{b}\right)$ as ${\hat{\theta }}_{k}$, we then have,

(8) $$\begin{array}{l} \sin{\hat{\theta }}_{k}\cdot \sin3{\hat{\theta }}_{k}=0\\ \cos{\hat{\theta }}_{k}\cdot \cos3{\hat{\theta }}_{k}=0 \end{array}$$

It can be easily proven that Equation (8) has no solution, suggesting that the method described in Figure 4 cannot achieve a steady state when motor is at a standstill. Therefore, ANF is obviously not suitable for applications where motors operate in a stationary state. Moreover, the slow convergence rate of ANF could be a severe problem for a short-stroke linear motor, in that the motor might have already completed the whole stroke before the ANF algorithm converges to a steady state.

Apart from ANF, the signal processing method based on SFE also has the abovementioned problems, which will not be discussed in detail in this section. In order to obtain signals without harmonics promptly, precisely and stably, EKF-based signal processing is proposed in this paper.

## 4. Signal Processing Using EKF

The EKF is a linearized extension of the Kalman filter. The Kalman filter has proven to be the optimal filter in a linear system, so the EKF also has merits such as fast convergence rate and high robustness. The EKF is a well-proven and commonly used recursive algorithm for signal estimation. Here, we use the EKF to estimate the third-order harmonic component based solely on the raw data measured by linear Hall sensors.

To employ the EKF, the state model and the observation model are first constructed. Taking the amplitude of the fundamental component and the percentage of the harmonic component as state variables, the two original signals detected by Hall sensors as outputs, and the raw electrical angle calculated by Equation (4) as input, variables used in the EKF model are represented as follows:

(9) $$x_{k}=\left[\begin{array}{c} u_{mk}\\ r_{k} \end{array}\right],y_{k}=\left[\begin{array}{c} u_{ak}\\ u_{bk} \end{array}\right],u_{k}={\hat{\theta }}_{k}=\mathrm{atan}2\left(u_{ak},u_{bk}\right)$$

*r_k_* is only related to the mechanical structure of the PMTLM, and *u_mk_* barely changes if the running condition of the PMTLM is unchanged. Hence, we can consider that *x_k+_*_1_ = *x_k_*, and then the EKF model can be described as in Equation (10).

(10) $$\begin{array}{c} x_{k+1}=I\cdot x_{k}+w_{k}\\ y_{k}=h\left(x_{k},u_{k}\right)+v_{k}\\ h\left(x_{k},u_{k}\right)=\left[\begin{array}{c} u_{mk}\left(\sin\theta_{k}-r_{k}\sin3\theta_{k}\right)\\ u_{mk}\left(\cos\theta_{k}+r_{k}\cos3\theta_{k}\right) \end{array}\right] \end{array}$$

where *w_k_* is the state equation noise, *v_k_* is the observation equation noise, and their covariance matrices are represented as *Q_k_* and *E_k_* respectively.

Employing Equation (5), we have

(11) $$\begin{array}{ll} h\left(x_{k},u_{k}\right) & \approx \left[\begin{array}{c} u_{mk}\sin\left({\hat{\theta }}_{k}+r_{k}\sin4{\hat{\theta }}_{k}\right)-u_{mk}r_{k}\sin3\left({\hat{\theta }}_{k}+r_{k}\sin4{\hat{\theta }}_{k}\right)\\ u_{mk}\cos\left({\hat{\theta }}_{k}+r_{k}\sin4{\hat{\theta }}_{k}\right)+u_{mk}r_{k}\cos3\left({\hat{\theta }}_{k}+r_{k}\sin4{\hat{\theta }}_{k}\right) \end{array}\right]\\ & =\left[\begin{array}{c} u_{mk}\sin\left(u_{k}+r_{k}\sin4u_{k}\right)-u_{mk}r_{k}\sin3\left(u_{k}+r_{k}\sin4u_{k}\right)\\ u_{mk}\cos\left(u_{k}+r_{k}\sin4u_{k}\right)+u_{mk}r_{k}\cos3\left(u_{k}+r_{k}\sin4u_{k}\right) \end{array}\right] \end{array}$$

The recursive EKF algorithm is divided into time prediction and measurement update [21,22]. Equations for them are as follows.

Time prediction:

(12) $${\hat{x}}_{k+1}^{-}=F_{k}\cdot {\hat{x}}_{k}$$

(13) $$P_{k+1}^{-}=F_{k}P_{k}F_{k}^{T}+Q_{k}$$

Measurement update:

(14) $$K_{k}=P_{k}^{-}H_{k}^{T}{\left(H_{k}P_{k}^{-}H_{k}^{T}+E_{k}\right)}^{-1}$$

(15) $${\hat{x}}_{k}={\hat{x}}_{k}^{-}+K_{k}\left(y_{k}-H_{k}{\hat{x}}_{k}^{-}\right)$$

(16) $$P_{k}=\left(1-K_{k}H_{k}\right)P_{k}^{-}$$

where *F_k_* is the identity matrix in our case, and *H_k_* is the linear approximation of *h (x_k_*, *u_k_*), i.e.,

(17) $$\begin{array}{l} H_{k}=\frac{\partial h}{\partial x}=\left[\begin{array}{cc} H_{11k} & H_{12k}\\ H_{21k} & H_{22k} \end{array}\right]\\ H_{11k}=\frac{\partial u_{ak}}{\partial u_{mk}}=\sin{\tilde{\theta }}_{k}-r_{k}\sin3{\tilde{\theta }}_{k}\\ H_{12k}=\frac{\partial u_{ak}}{\partial r_{k}}=u_{mk}\cos{\tilde{\theta }}_{k}\sin4u_{k}-u_{mk}\sin3{\tilde{\theta }}_{k}-3u_{mk}r_{k}\cos3{\tilde{\theta }}_{k}\sin4u_{k}\\ H_{21k}=\frac{\partial u_{bk}}{\partial u_{mk}}=\cos{\tilde{\theta }}_{k}+r_{k}\cos3{\tilde{\theta }}_{k}\\ H_{22k}=\frac{\partial u_{bk}}{\partial r_{k}}=-u_{mk}\sin{\tilde{\theta }}_{k}\sin4u_{k}+u_{mk}\cos3{\tilde{\theta }}_{k}-3u_{mk}r_{k}\sin3{\tilde{\theta }}_{k}\sin4u_{k}\\ {\tilde{\theta }}_{k}=u_{k}+r_{k}\sin4u_{k} \end{array}$$

Based on Equations (12)–(17), *u_mk_* and *r_k_* are estimated online. Then, the third-order harmonics can be calculated and removed. We have the following:

(18) $$\begin{array}{c} {\bar{u}}_{ak}=u_{ak}+u_{mk}r_{k}\sin(3{\hat{\theta }}_{k})\\ {\bar{u}}_{bk}=u_{bk}-u_{mk}r_{k}\cos(3{\hat{\theta }}_{k})\\ {\bar{\theta }}_{k}=\mathrm{atan}2\left({\bar{u}}_{ak},{\bar{u}}_{bk}\right) \end{array}$$

where ${\bar{u}}_{ak},{\bar{u}}_{bk}$ are the estimated fundamental components and ${\bar{\theta }}_{k}$ is the estimated electrical angle.

In this way, the signals after EKF processing no longer contain large third-order harmonics. Small errors may still exist due to the approximation error in Equation (5), but most of the harmonics are eliminated, contributing to a more precise mover position detection result.

**Figure 5:**
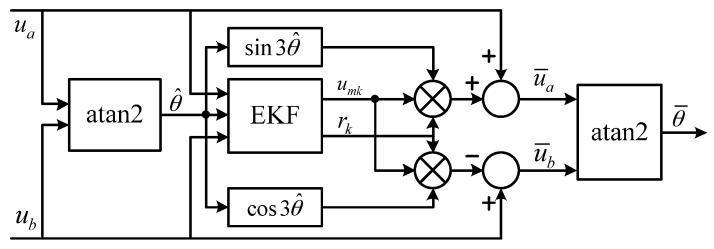
Diagram of EKF processing.

The diagram of EKF processing is shown in Figure 5. The estimated electrical angle $\bar{\theta }$ is not feedback to the signal processing procedure. Instead, the raw electrical angle $\hat{\theta }$ is used for EKF processing: $\left({\hat{\theta }}_{k}+r_{k}\sin4{\hat{\theta }}_{k}\right)$ rather than $\left({\hat{\theta }}_{k}+r_{k}\sin4{\bar{\theta }}_{k}\right)$ is used as the input of EKF, and $\sin3{\hat{\theta }}_{k}$ rather than $\sin3{\bar{\theta }}_{k}$ is used to produce the reference of third-order harmonic component. There are two reasons for doing so. First, the difference is small between ${\hat{\theta }}_{k}$ and ${\bar{\theta }}_{k}$, and it becomes even smaller between $\sin n{\hat{\theta }}_{k}$ and $\sin n{\bar{\theta }}_{k}$, thus the adverse impact of this approximation is limited. In addition, as long as *u_mk_* and *r_k_* stay around the true values, $\bar{\theta }$ will stick to the true value. When the motor operates at zero speed, the input and output data of EKF remain unchanged, and then they can be considered useless data. The useless data have no effect on the update of state variables, so the convergence process of EKF is not undermined. Therefore, useless data will not cause the divergence of *u_mk_* and *r_k_*, and then the stability of EKF processing at zero speed is guaranteed.

## 5. Experimental Results

**Figure 6:**
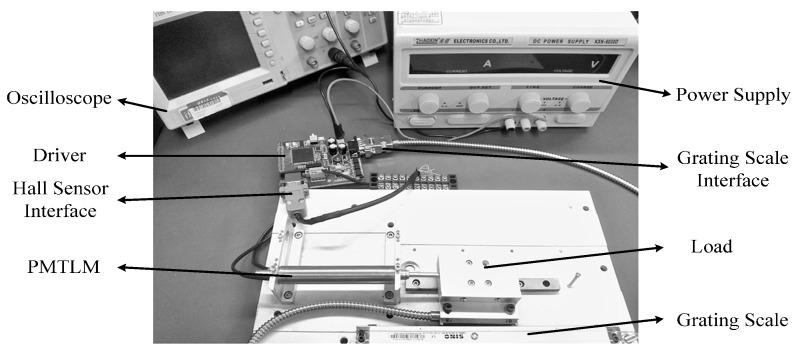
Experimental platform.

In this section, extensive experimental results are provided to confirm the validity of the proposed method. The experimental platform is shown in Figure 6. A grating scale is installed to provide the true value of the mover position, and its resolution is 1 μm. The motor driver adopts a voltage source inverter, and the switching frequency is 10 kHz. A float-point digital signal processor (DSP) TMS320F28335 is used to run the position detection and servo control algorithm. In order to deal with the high nonlinearity of the model described in Equation (10), suboptimal multiple fading factors are employed in Equation (13) of EKF processing in actual experiments, and this can significantly enhance the robustness against system nonlinearity and model uncertainty [23,24].

**Figure 7:**
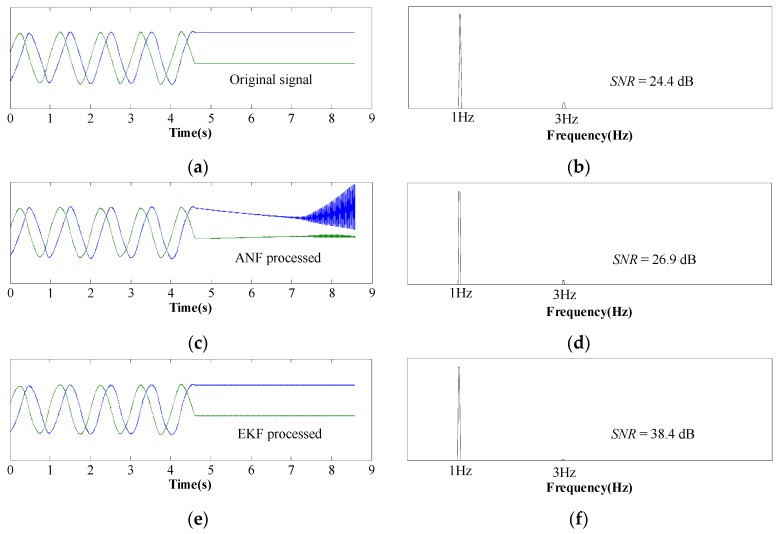
Waveforms and FFT results of sensor signals at 20mm/s. (**a**,**b**) Original sensor signals. (**c**,**d**) ANF processed signals. (**e**,**f**) EKF processed signals.

Figure 7 describes the waveforms and FFT results of Hall sensor signals at the speed of 20 mm/s. The motor is at a standstill after 4.5 s. Apparently, the two Hall sensors output sine and cosine signals when the motor is moving and output constant signals when the mover stops moving. The FFT result of sine/cosine waves is depicted in Figure 7b, which indicates that third-order harmonics exist in the original sensor signals. ThenANF-based signal processing method is first utilized to remove third-order harmonics, and the corresponding FFT result shows that the harmonic content is reduced. However, the ANF shows instability at zero speed, meaning that the ANF processed signals diverge from the original signals. Conversely, EKF processed signals remain stable when the mover is at a standstill, as depicted in Figure 7e. Moreover, Figure 7f shows that the harmonic content is further reduced when sensor signals are processed by the EKF, meaning that the EKF has a better filtering performance than the ANF.

**Figure 8:**
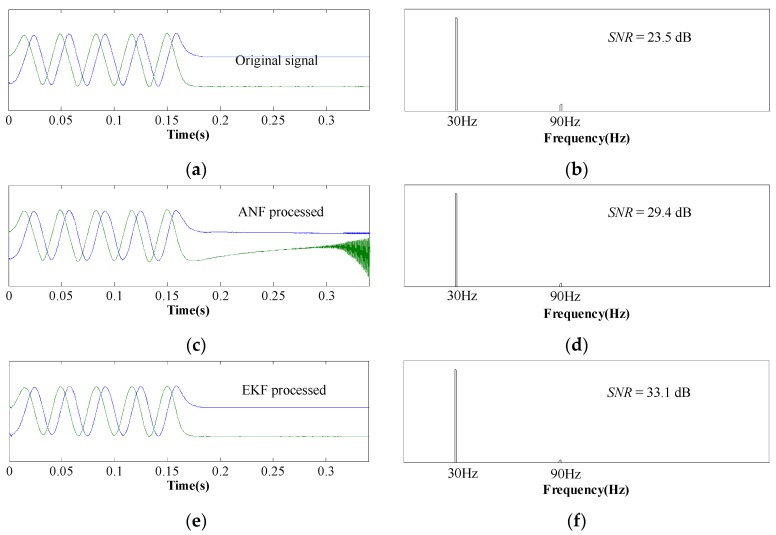
Waveforms and FFT results of sensor signals at 600 mm/s. (**a**,**b**) Original sensor signals. (**c**,**d**) ANF processed signals. (**e**,**f**) EKF processed signals.

Figure 8 shows experimental results at the speed of 600 mm/s, and the waveforms and FFT results are consistent with Figure 7. Therefore, we can come to the conclusion that EKF processing is able to minimize the third-order harmonics at different speeds, including zero speed.

**Figure 9:**
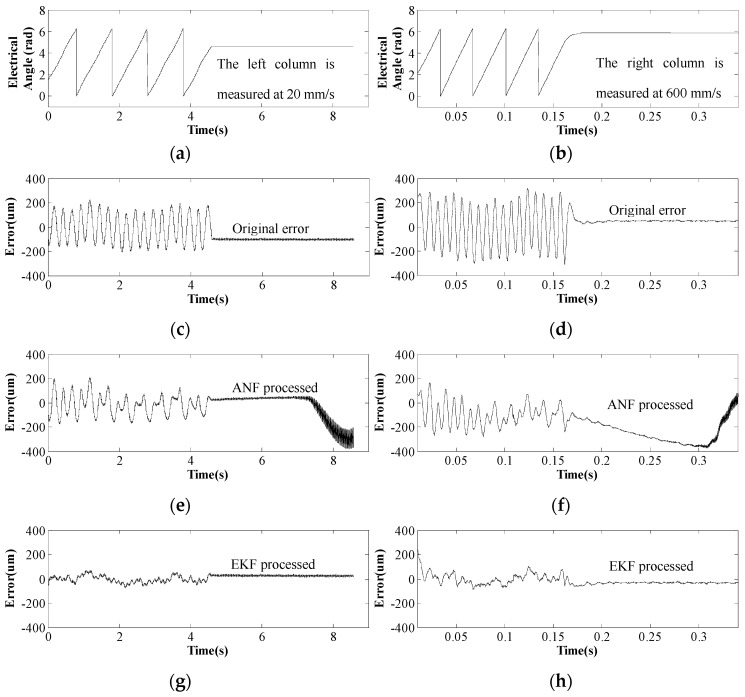
Position detection error at 20 mm/s and 600 mm/s. (**a**,**b**) Electrical angle of PMTLM. (**c**,**d**) Position detection error when using original sensor signals. (**e**,**f**) Error when using ANF processed signals. (**g**,**h**) Error when using EKF processed signals. The left column is measured at 20 mm/s, and the right column is measured at 600 mm/s.

When the processed signals are used for position calculation, the performance of different processing methods can be more specifically observed from Figure 9. Figure 9c,d demonstrate the original error of position detection when using raw sensor data. It can be seen that the maximum error is about 200 μm, and the original error fluctuates four times during an electrical period, which agrees with Equation (5). In order to reduce this fourth-order error in position detection results, the third-order harmonics in Hall sensor signals are supposed to be filtered out. When ANF is applied, its position detection error is described in Figure 9e,f. The error begins to diminish at the second electrical period, and yet diverges when the mover speed drops to zero. Compared with ANF, EKF can converge within several signal sampling periods, thus EKF processing can minimize the error more rapidly and more effectively. When EKF processing is employed, the maximum error is reduced to 60 μm at 20 mm/s and 100 μm at 600 mm/s (see Figure 9g,h). Furthermore, the error sticks to zero when PMTLM operates at zero speed, which is consistent with discussions in Section 4. Apparently, EKF processing shows better performance than ANF processing for PMTLM mover position detection.

**Figure 10:**
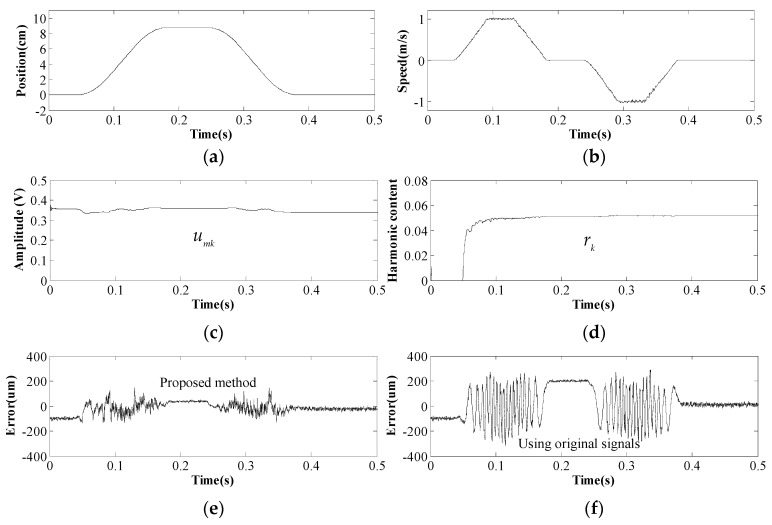
Position control of PMTLM using proposed position detection method. (**a**) Mover position of PMTLM; (**b**) Mover speed of PMTLM; (**c**) Convergence process of *u_mk_*; (**d**) Convergence process of *r_k_*; (**e**) Position detection error using proposed method; (**f**) Position estimation error using original sensor signals.

Finally, the mover position detected by the proposed method is used for closed-loop control of the PMTLM, and the driver system operates well, as shown in Figure 10. The mover gradually marches to 90 mm at a top speed of 1 m/s, halts for 50 ms, and then moves backward to the origin.

The convergence process of EKF is depicted in Figure 10c,d. Both *u_mk_* and *r_k_* stay stable in all speed regions and remain invariant at zero speed. Obviously, sensor signals measured at zero speed would not update the state variables of EKF, and this explains why *r_k_* starts to converge at 0.05 s. Also, the value of *u_mk_* fluctuates slightly along with the mover position, and this suggests that the magnetic field strengths of different PMs are distinctive, which could be caused by manufacturing differences.

It can be seen in Figure 10e that the dynamic error of the proposed position detection method is within 100 μm, which is much smaller than the position estimation error using the original Hall sensor signals (see Figure 10f). On the other hand, it is also noteworthy that the error at zero speed is significantly reduced from 200 μm to 36 μm. Therefore, the presented method can achieve both higher dynamic accuracy and higher positioning accuracy. Even though there are still errors due to things like sensor error or mechanical error, the proposed EKF processing method seems to be feasible and superior.

## 6. Conclusions

Linear Hall sensors are utilized for mover position detection of PMTLM, and the third-order harmonics in Hall sensor signals seriously impact the accuracy. In this paper, an EKF-based signal processing method is introduced to filter out harmonics. The proposed method is compared with the conventional method and validated by substantial experiments. In the end, a PMTLM control loop based on EKF processing is constructed to manifest its performance in whole speed region. Unlike the conventional method, EKF processing can converge within several sampling periods, and the processed signals remain stable at a standstill, so both the convergence rate and the stability are enhanced. Furthermore, the proposed method also has higher position detection accuracy, whether the mover is dynamic or static. The proposed method has the advantages of low cost, small volume and high accuracy, and can be easily extended to rotary machines and signals containing higher order harmonics. The EKF processing algorithm can also be used for sensorless control methods to eliminate harmonics in the observed back-EMF.
